# The Physical Changes Produced by Decay

**Published:** 1890-01

**Authors:** 


### The Physical Changes Produced by Decay.

BY FELIX.

The theories advanced to account for the changes produced by decay are two in number—the chemical theory and the germ theory.

The chemical theory accounts for these changes through the action of acids upon the inorganic and organic constituents of the teeth. Dr. Watt's theory gives clearly the characteristics of white, brown and black decay as produced by each respectively of the three mineral acids, nitric, hydrychloric and sulphuric, accounting for the formation of these acids by purely chemical reactions, and accounting for their action upon the teeth in the same manner.

Dr. Magitot, while acknowledging the undoubted action of these acids upon the teeth, proves by direct experiment the exceedingly injurious action of many vegetable acids, salts and albuminoids, accounting for these partly by the chemical theory of acid reactions and partly by the germ theory of fermentative processes.

Dr. Miller has developed the germ theory in connection with the fermentative processes until he has proved that the saliva, normal, healthy saliva, contains an organized ferment which upon coming into contact with sugar or starch causes acid fermentation. This fermentative process in the presence of sugar or starch develops an acid, which Dr. Miller believes to be the direct cause of decay.

Thus, in brief, we have the two principal theories of decay, both accounting for the changes produced in the normal structure of the teeth by the reaction of acids, for the action of fermentation is through the production of an acid, the germ acting only in this capacity as far as its action is related to the tooth.

The sum and result of this conclusion is that the physical changes produced by decay are the same by whatever theory we may acconnt for their production, so that the object of this paper is simplified and rendered clear by the fact that we have to do with the results of the action of various acids upon the different parts of the teeth.

The physical changes produced by the three mineral acids most actively concerned in ordinary decay will be briefly noticed first. Nitric acid acts with energy upon all the constituents of the teeth, dissolving so promptly that only a slight residue remains to indicate the agent producing the decay. It dissolves the inorganic salts early in the carious cavity, producing softening of the dentine preceding the complete destruction of the tissue. The residue is white in color and friable in composition.

Hydrochloric acid acts more energetically upon the inorganic portion of the tooth, usually leaving the organic matter in the form of a brown, leathery deposit in several layers and of considerable thickness. Both nitric and hydrochloric acids dissolve the phosphate of lime and decompose the carbonate, setting the carbonic acid free.

Sulphuric acid acts principally upon the organic portion of the tooth, carbonizing it and coagulating the albumen, thus producing the black decay, which progresses only slowly, inasmuch as the acid is unable to dissolve the phosphate of lime.

Beside these ordinary conditions of decay we have several kinds from Dr. Magitot's experiments, principal among which are the vegetable acids. Lactic, butyric, citric and malic acids produce similar changes with each other, acting upon all the tissues of the tooth, destroying the organic and inorganic portions completely, and during the progress of the decay leaving the cavity in a blackened condition. Alum and bioxalate of potassa may be mentioned as acting very energetically on the enamel and not at all upon the cementum or dentine, dissolving the substance of the enamel rapidly but stopping at the dentine. This action is not as yet understood.

Acetic and tartaric acids and acid tartrates act upon the dentine and cementum but not upon the enamel, dissolving with energy the phosphates found in all tissues, but prevented from attacking the enamel probably by the chemical and vital bonds keeping the phosphate from decomposing.

Albumen and the albuminoids do not act directly upon the teeth, but they ferment in the mouth and act upon other substances as ferment, producing agents thus resulting in fermentation and formation of acids, lactic, butyric, valeric, and so on, all of which are directly concerned in decay. Sugar in any form will be fermented in the mouth under the ordinary conditions of want of care of the teeth, and especially the condition of carious teeth. This produces lactic and butyric acids and is equivalent to acid decay, with this difference, viz.: that the acids attack the tooth while in a nascent condition.

By the germ theory caries of the dentine and enamel has been produced by placing in contact with the teeth sugar together with some nitrogenous material and the fungus from carious dentine. Thus this fungus is proven to be the active agent in caries as without it the decay does not occur. The action of the fungus is however in producing fermentation, and thus acid, thus simplifying the problem of the physical changes down to the action of acids.

Having thus considered briefly the action of the various agents concerned in caries, let us consider the resistance offered by the tooth, and then balancing the constructive and destructive forces acting upon the tooth arrive at a correct conclusion as to the actual changes produced by decay.

The constructive forces are mainly concerned in the formation of new tissue and in solidifying the already formed dentine. This is occasioned by the excitation of the pulp caused by the irritation of the dentine from the progress of the decay. Increased nutriment is furnished the dentine which becomes more dense, and the tubules are finally obliterated except that a few straight tubules remain open of large size usually. This deposit of dentinal matter is found in the tubules leading from the decayed surface to the pulp, forming a cone of resistance, thus opposing to the progress of the decay an almost solid zone of compact tissue with hardly any openings for ingress of foreign matter.

This retards decay and often effectually checks it. If, however, decay progresses in spite of this resistance, and approaches near the pulp, the formation of dentine extends into the pulp chamber, forming sometimes only a nodule capping the apex of the resistance cone sometimes spiculæ in different parts of the organ itself, and in extreme cases of decay filling the entire pulp chamber, obliterating the upper portion of the pulp and opposing a solid mass of secondary dentine. Sometimes the decayed layer seems as a protection to the normal dentine beneath, as in sulphuric acid decay; sometimes, however, the decayed layer is a place for chemical changes where acid is constantly being evolved.

Now, considering these opposing forces, the progress of decay is measured by their relative strength. In weak constitutions and slight nutritive force the vital resistance is weak, the progress of the disease unchecked, and therefore rapid when the condition of the mouth is such as to favor decay. On the other hand, in the case of a strong constitution and vigorous nutritive force the resistance is great and caries progresses only slowly even if the condition of the mouth is such as to favor it. The contest between these opposing forces is greatly affected also by the normal or abnormal condition of the teeth, as induced, by the conditions under which they were developed.

Following this chain of reasoning we find a great variety of cases in actual practice. In one case the pulp is unable to protect itself but becomes soon exposed with no formation of secondary dentine at all; the pulp dies with only slight pain or none at all; and very slight exposure, or none at all; the root becomes inflamed and abscess forms while the patient is unaware even of decay.

In another case as decay progresses, secondary dentine forms rapidly and the dentine rendered impervious; caries is checked, and if general systemic conditions are favorable may remain in this condition for a long time.

Still another case occurs where secondary dentine forms rapidly, or even slowly, keeping the pulp protected securely while decay has enough force to constantly progress nearer and nearer the pulp. The pulp obliterates itself, however, gradually until the excavation is carried to the limits of the portion of the pulp chamber in the crown of the tooth. In both of these cases the trifacial nerve may not be irritated so as to cause any severe pain, and reparation by filling may be easily applied.

In regard to pain, however, the first case may be markedly different; the slightest exposure of the dentine without corresponding formation of secondary dentine may cause acute pain, and even neuralgia. One of the physical changes caused by irritation of the dentine and pulp is found in inflammatory conditions of the pulp; this may lead to entire death of the organ and through the apical foramen abscess in the jaw bone. Chronic abscess may cause necrosis of the bone as a direct result of decay of the teeth.

As an indirect result of decay of the teeth we have tumid gums caused by cavities existing below the margin of the gums and irritating them. Farther than this the gums may develop a polypus or a simple epulis may develop into a malignant growth. The direct irritation from the sharp margin of a decayed cavity has been the cause of epithelioma; while several cavities in the mouth have been the direct cause of indigestion through the impure debris which they contain.

